# Weather Variability Associated with *Aedes (Stegomyia) aegypti* (Dengue Vector) Oviposition Dynamics in Northwestern Argentina

**DOI:** 10.1371/journal.pone.0127820

**Published:** 2015-05-20

**Authors:** Elizabet L. Estallo, Francisco F. Ludueña-Almeida, María V. Introini, Mario Zaidenberg, Walter R. Almirón

**Affiliations:** 1 Centro de Investigaciones Entomológicas de Córdoba, Instituto de Investigaciones Biológicas y Tecnológicas (IIBYT), CONICET-Universidad Nacional de Córdoba, Facultad de Ciencias Exactas, Físicas y Naturales. Av. Vélez Sarsfield 1611, Ciudad Universitaria, CP, X5016GCA, Córdoba, Argentina; 2 Ministerio de Salud de la Nación, Coordinación Nacional de Control de Vectores, Buenos Aires, Argentina; University of Thessaly, GREECE

## Abstract

This study aims to develop a forecasting model by assessing the weather variability associated with seasonal fluctuation of *Aedes aegypti* oviposition dynamic at a city level in Orán, in northwestern Argentina. Oviposition dynamics were assessed by weekly monitoring of 90 ovitraps in the urban area during 2005-2007. Correlations were performed between the number of eggs collected weekly and weather variables (rainfall, photoperiod, vapor pressure of water, temperature, and relative humidity) with and without time lags (1 to 6 weeks). A stepwise multiple linear regression analysis was performed with the set of meteorological variables from the first year of study with the variables in the time lags that best correlated with the oviposition. Model validation was conducted using the data from the second year of study (October 2006- 2007). Minimum temperature and rainfall were the most important variables. No eggs were found at temperatures below 10°C. The most significant time lags were 3 weeks for minimum temperature and rains, 3 weeks for water vapor pressure, and 6 weeks for maximum temperature. *Aedes aegypti* could be expected in Orán three weeks after rains with adequate min temperatures. The best-fit forecasting model for the combined meteorological variables explained 70 % of the variance (adj. R^2^). The correlation between *Ae*. *aegypti* oviposition observed and estimated by the forecasting model resulted in *r_s_* = 0.80 (P < 0.05). The forecasting model developed would allow prediction of increases and decreases in the *Ae*. *aegypti* oviposition activity based on meteorological data for Orán city and, according to the meteorological variables, vector activity can be predicted three or four weeks in advance.

## Introduction

*Aedes (Stegomyia) aegypti* is well known as the main vector of dengue [1], widely distributed from tropical to subtropical areas of the world [2]. In Argentina, it reaches central temperate regions of the country [3–5], although eggs have also been found in the Patagonia region (Neuquén Province) [6].

Dengue is a major public health concern throughout tropical and subtropical regions of the world. It is the most rapidly spreading mosquito-borne disease [2], affecting populations of all ages and socio-economic levels, with an estimated 2.5 billion people living in countries at-risk, and 50 to 100 million cases per year. Dengue incidence has increased 30-fold in the Americas in the last 50 years, and between 2008–2012 more than 1.2 million cases of dengue were notified annually, including 28,233 severe cases and 1,000 deaths [7]. Furthermore, 2013 had the largest epidemic in the history of the Americas. The burden of disease was the highest ever registered, with a total of 2.3 million cases, 37,898 severe cases and 1,318 deaths [8].

Dengue outbreaks in the warm northern part of Argentina are directly associated with occurrence of the virus circulating in neighboring countries (Paraguay, Brazil and Bolivia) [9]. A major dengue outbreak in 2009 reached temperate regions of central Argentina, including large cities such as Buenos Aires [10] and Córdoba [4], although 92% of cases were reported in the northern provinces of Chaco (46%), Catamarca (36%) and Salta (10%) [11].

According to the World Health Organization, research will continue to play an important role in reversing the trend in dengue, a neglected tropical disease, by improving methods and systems for surveillance, prevention and control [2]. Therefore, regional specific studies of population dynamics are fundamental considering that populations of *Ae*. *aegypti* may show variations in behavior in different geographical areas [12–15]. Thus, seasonal studies of oviposition activity and patterns of abundance of the vector are necessary to predict periods of higher risk of virus transmission [9].

Few studies attempting to develop a forecasting warning system have been carried out in northwestern Argentina. In our previous studies we have constructed statistical models to forecast larval indices (House and Breteau indices) based on environmental data obtained from satellite images and meteorological variables in Oran [16,17]. Mathematical models of *Ae*. *aegypti* oviposition activity allowed us to determine the most effective timing for vector control measures, considering its intrinsic rate of population growth [18]. A study of spatial patterns of high *Ae*. *aegypti* oviposition activity has also been developed for Oran, which enabled a predictive map with areas of maximum probability of *Ae*. *aegypti* oviposition activity and high risk areas of transmission [5].

In temperate areas of Argentina, studies have dealt with *Ae*. *aegypti* oviposition dynamics and stochastic population dynamic models [3,13,19,20]. These studies have generated tools for vector surveillance in the region.

Dengue outbreaks can be reduced by implementing improved prediction models, and through coordinated epidemiological and entomological surveillance, promoting the principles of integrated vector management and deploying locally-adapted vector control measures [2]. Here, we develop a forecasting model to assist vector control programs by assessing the weather variability associated with seasonal fluctuation of *Ae*. *aegypti* oviposition dynamics at a city level in San Ramón de la Nueva Orán (Salta Province), northwestern of Argentina.

## Material and Methods

### Study area

**Fig 1:**
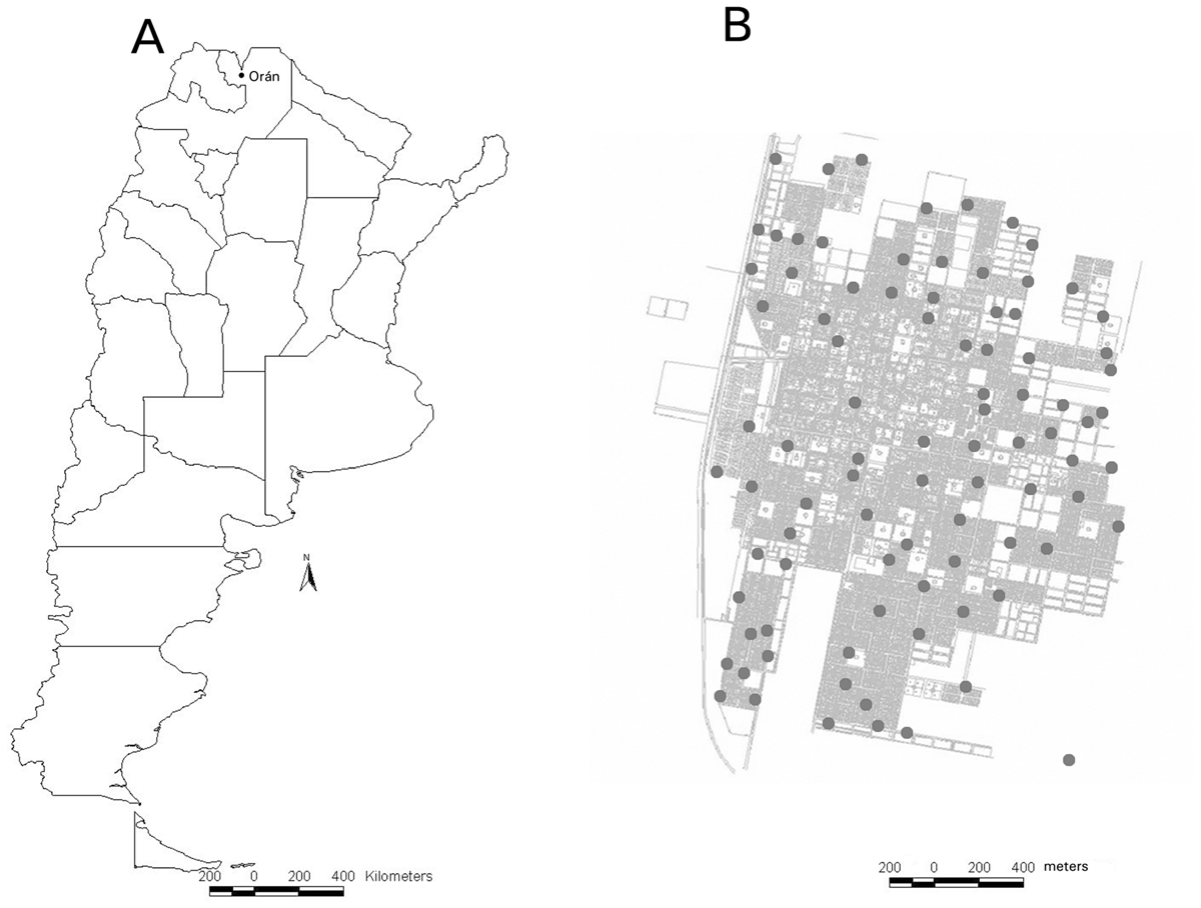
Study site. A: Map of Argentina showing in Salta province (northwestern) the study locality. B: Map of the study locality: San Ramón de la Nueva Orán (4 km X 6 km extension) indicating where the 90 ovitrap were placed. The sampling points were used in spatial patterns of high *Aedes aegypti* oviposition activity published in an earlier paper (5).

This study took place in Orán in a subtropical region of the northwest Argentina (23° 08' S, 64° 20' W) (Fig 1). Orán (336 m.a.s.l) has 82,413 inhabitants [21], including 76,070 in the urban area of the city. Orán is the second largest urban center of the province, located 270 km north of the provincial capital of Salta. Orán is an important urban center, and trades goods with Bolivia, 50 km to the north. City maximum and minimum absolute temperatures are 44.5°C and 11.5°C in summer and 38.9°C and -3.6 in winter respectively. One or 2 frosts (from 3 to 5 hours of duration) occur in winter (July). Frost frequency, as well as intensity and duration, are variables in the area [22]. During the winter of 2007, the frosts were the most intense, since 1978, resulting in the declaration of a crop emergency in the area [23]. The mean annual rainfall is over 1000 mm and the mean relative humidity is 78% [24].

### Ovitraps sampling

Seasonal fluctuation of *Ae*. *aegypti* oviposition was assessed by weekly ovitrap sampling in the urban area of the city, from the last week of October 2005 to the last week of October 2007. Each ovitrap consisted of 350 ml transparent plastic jar (9 cm high and 8 cm in diameter), lined with a cylinder of brown heavy weight (120 g) filter paper [25, 26, 27]. The paper was cut to fit the jars snugly, with an overlap to cover the entire interior surface of each jar. Ovitraps were filled with 250 ml of grass infusion (dry grass macerated in regular water for one week). This infusion results in an effective attractant to gravid *Ae*. *aegypti* female mosquitoes [25, 26]. Orán was divided into 3 areas (north, central and south) and from each area 30 points were randomly selected with the use of a random number table (N = 90 houses). Ovitraps were placed outdoors (gardens or backyards) in a shade area, located at floor level or at a maximum height of 50 cm in private residences, whose owners previously provided permission to carry out the study (Fig 1). Ovitraps were replaced weekly and the number of eggs of each were counted with a 10x magnifying glass [26] and recorded. All houses were successfully monitored throughout the entire study.

### Meteorological variables

Daily meteorological data (rainfall, photoperiod, water vapor pressure, temperature and relative humidity maximum/minimum) were provided during the sampling period by the Orán office of the Meteorological National Service (SMN), and weekly averages were calculated for each variable (except rainfall, for which accumulated values were used) over the study period. The only weather station was located in the south of the city (23° 09' 17.03'' S, 64° 19' 41.50'' W), and its data reflected the conditions of the entire city.

### Data analysis

Simple linear correlation analysis was performed, using Spearman’s rank correlation coefficient (*r*_*s*_), between the weekly number of eggs and the rainfall average (mm), photoperiod, water vapor pressure (Hpa), temperature (°C) and relative humidity (%). Correlations were performed with and without time lags from 1 to 6 weeks. Correlations were performed using the InfoStat/Profesional statistical software [28]. To develop a forecasting model, a multiple linear regression analysis (stepwise backward elimination) was performed with the set of meteorological variables from the first year of study (October 2005- September 2006) for the time lags that best correlated with the oviposition values. Initially, univariate models were also tested, but to develop the model, it was necessary to include several variables. Stepwise backward elimination led to a parsimonious forecasting model (fewer variables but with a significant forecasting model). Model validation used data from the second year of study (October 2006-September 2007). The model included the meteorological variables with those time lags that best correlated with the registered number of laid eggs. The number of eggs (*N*) was transformed into *Ln (N+1)* to satisfy normality and variance homogeneity assumptions. Validating the model, the data set of the regression variables corresponding to the second year were used as entries to the developed model. Finally, the Spearman’s rank correlation between the values observed in the second sampling year and those estimated by the model was calculated. The model was developed using the R3.1.0 software [29]. In addition, the multicollinearity test of the regression variables was examined by means of the variance inflation factor (VIF) for each variable included in the model. The AIC (Akaike Information Criterion) was used to identify the most parsimonious model, corresponding to the model with the lowest AIC [30]. The ovitrap data used in the current study for the development of the predictive model have been published and spatially analyzed before in an earlier study [5].

## Results

### Oviposition patterns

**Fig 2:**
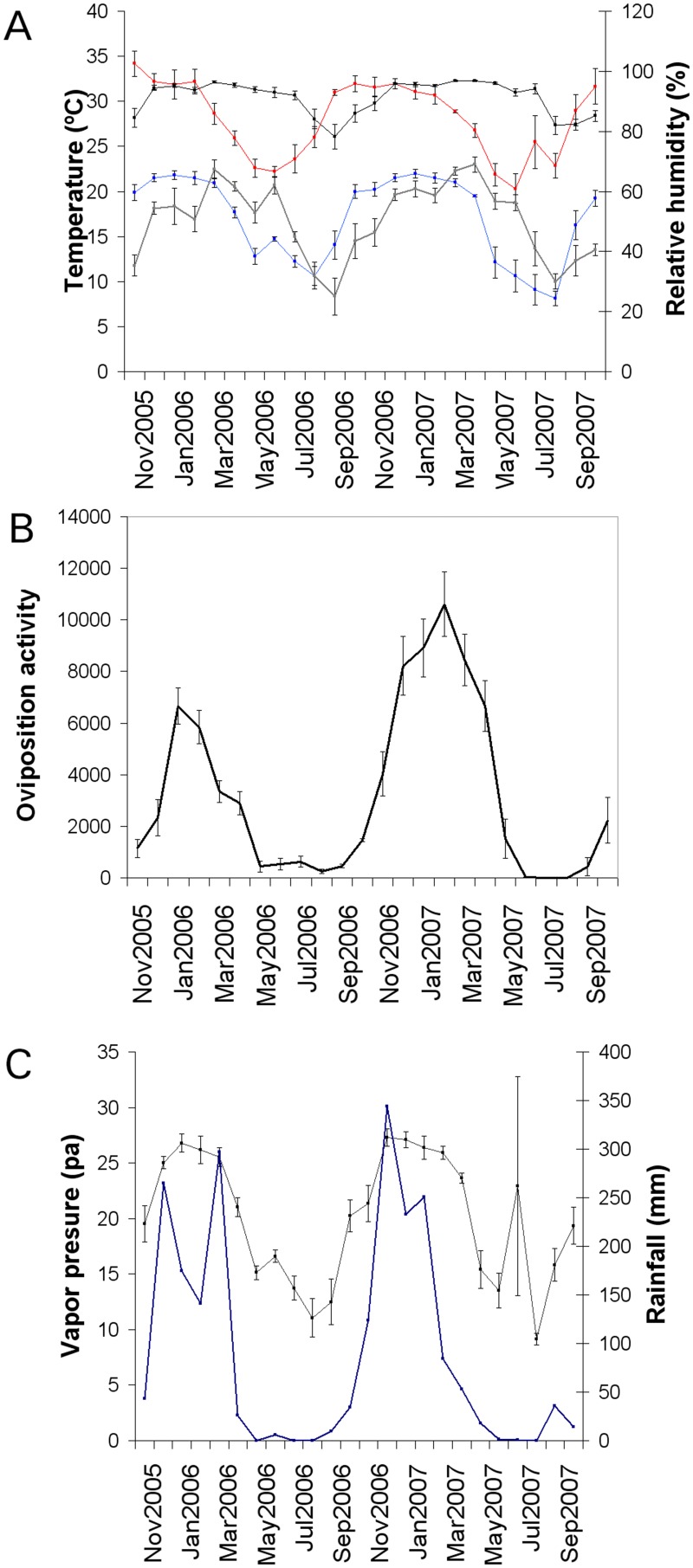
Monthly average meteorological data and oviposition activity from Orán city (November 2005 to October 2007). A: minimum temperature (blue), maximum temperature (red) and minimum and maximum relative humidity (black) (average values ±SE). B: Observed oviposition activity of *Ae*. *aegypti*. C: water vapor pressure (black) (average values ±SE) and accumulated monthly rainfall (blue).

A total of 321,141 *Aedes aegypti* eggs were detected during the 106 consecutive weeks, including 109,253 in the first year and 211,888 in the second year. During the summer months (December to March), 21.54% of the total eggs were collected the first year and 38.37% were collected in the summer of the second year. During January of both years, peaks of oviposition occurred, during periods with average maximum and minimum temperatures approximating 30°C and 21°C, respectively (Fig 2). Annual accumulated rainfalls in that period were normal, approximately 1,000 mm (Fig 2), and average maximum and minimum humidity approximated 95% and 60%, respectively (Fig 2).

### Meteorological variables and oviposition data

*Aedes aegypti* ovipositions were continuous during the first sampling year, but not for the second year. No eggs were found during 9 consecutive weeks of the second year (winter 2007- July to September). This was due to extremely low temperatures (Fig 2). During that period, the average minimum temperature per week was below the Developmental Thermal Threshold of 12.8°C [18] (Table 1).

**Table 1:** Oviposition activity and temperature between 13 June and 5 September 2007, in Orán.

Sampling date (weeks)	Egg number	Minimum weekly average temperature (°C)	Maximum weekly average temperature (°C)
Jun 13–19	57	12.0	20.4
Jun 20–26	0	11.9	19.7
Jun 27- Jul 3	22	5.5	22.8
Jul 4–10	0	9.6	18.8
Jul 11–17	0	2.0	19.4
Jul 18–24	0	7.0	24.3
Jul 25–31	0	4.9	22.1
Aug 1–7	0	6.2	18.7
Aug 8–14	0	9.2	24.3
Aug 15–21	0	9.8	19.9
Aug 22–28	0	9.2	23.5
Aug 29- Sep 4	0	9.6	28.4
Sep 5–11	213	19.3	33.8

Eggs were observed only in weeks 1 and 3. Data were recorded for each week between 4 July and 5 September.

Egg number: total number of eggs in 90 ovitraps during each time period.

Exceptional frosts were also registered on 11 and 12 July 11, 2007 (the absolute minimum temperature registered in those days was -0.8°C) (Table 1). According to the Meteorological National Service (SMN), the average maximum and minimum temperatures for July 1981–1990 were 21.8°C and 9.3°C, respectively. The mean maximum and minimum temperatures were 24.2°C and 12.3°C in July 2006, and 21.3°C and 6.0°C in July 2007. Examining the circumstances in winter 2007, the weekly average minimum temperature (9.77°C) occurred 3 weeks before the weeks in which no oviposition was detected (i.e., between June 13^th^ and July 4^th^, 2007). No eggs were found when the temperature fell below 10°C, which could represent a critical temperature and is the likely cause of the lack of oviposition.

All meteorological variables were significantly correlated with ovitrap data across time lags, excepting photoperiod (P> 0.05). Rainfall, temperature, humidity and vapor pressure of water fluctuated through time in a manner similar to *Ae*. *aegypti* oviposition activity (Fig 2). The most significant time lags were 3 weeks for minimum temperature and rains, 3 weeks for water vapor pressure, and 6 weeks for maximum temperature (Table 2).

**Table 2:** Meteorological variables and Spearman correlation: without and with time lag and the best correlation with the corresponding time lag (P < 0.05).

Independent variable	Correlation without time lag	Correlation with time lag	Time lags with best correlation (weeks)
*X*_1_- Maximum temperature	0.26	0.56	6
*X*_2_- Vapor pressure of water	0.76	0.61	2
*X*_3_- Minimum temperature	0.64	0.72	3
*X*_4_- Maximum humidity	0.32	0.32	2
*X*_5_- Minimum humidity	0.40	0.46	2
*X*_6_- Precipitation	0.41	0.62	3

**Fig 3:**
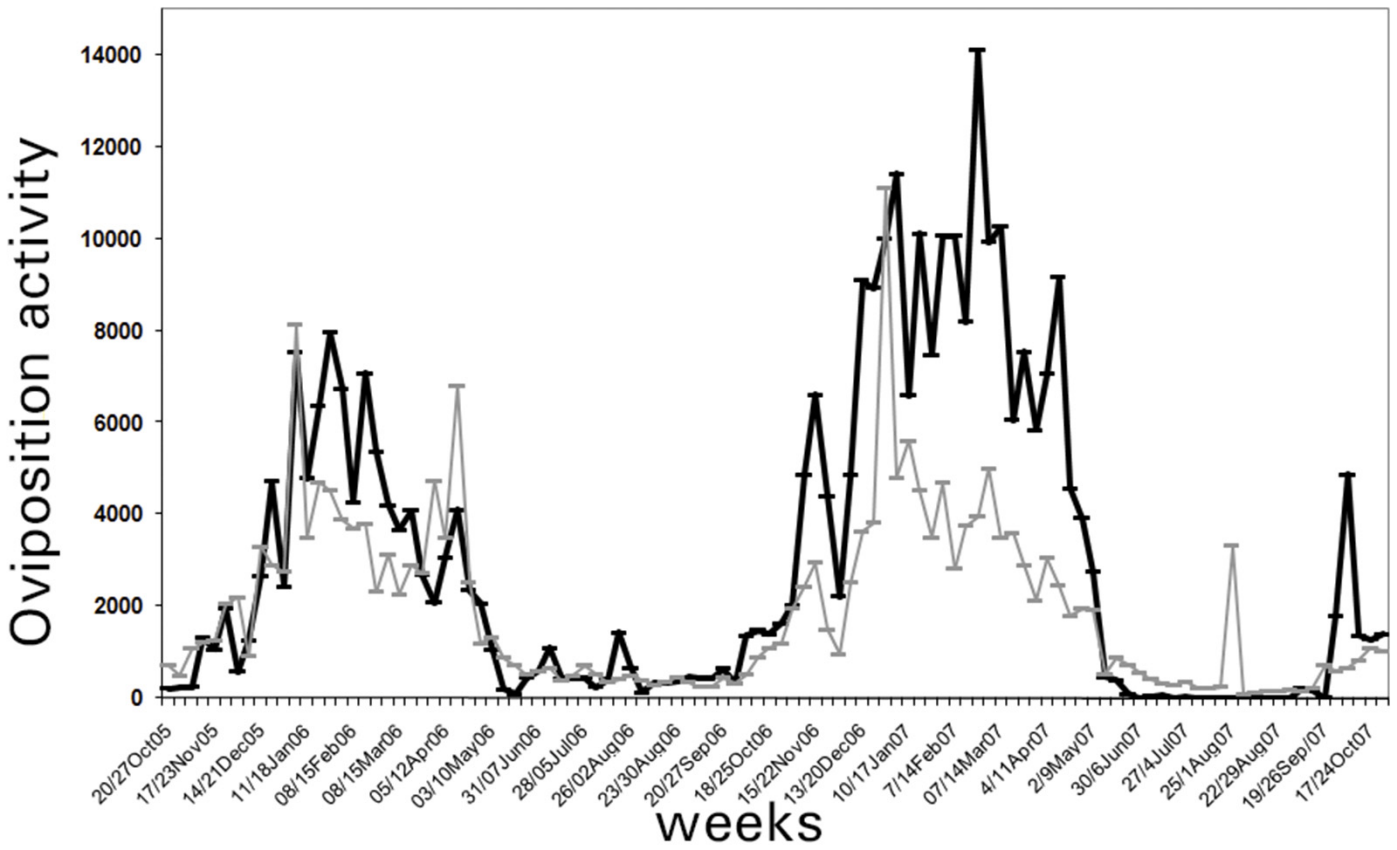
*Aedes aegypti* oviposition observed (black) and predicted (gray) activity during October 2005—October 2007 in Orán city, Argentina.

*Development of Ae*. *aegypti oviposition forecasting model*. The best-fit forecasting model for the combined meteorological variables accounted for 70% of the total sum of squares (adj. R^2^). Multicollinearity was not found (VIF < 4) and all time lag meteorological variables in the forecasting model were significant (Fig 3). Separate correlations for each prediction variable, with and without time lags, are summarized in Table 2. The composite forecasting model is as follows:

$$Ln\;\left(N+1\right){\;}_{\left(t\right)}=5.076+0.086\;X_{1}{}_{\left(t-6\right)}+0.063\;X_{2}{}_{\left(t-2\right)}+0.038\;X_{3}{}_{\left(t-3\right)}-0.035\;X_{4}{}_{\left(t-2\right)}+0.018\;X_{5}{}_{\left(t-2\right)}+0.006\;X_{6}{}_{\left(t-3\right)}$$

To validate the forecasting model, predicted values were calculated using meteorological variables of the second sampling year. The correlation (*r*_*s*_ *)* between *Ae*. *aegypti* oviposition observed and the *Ae*. *aegypti* oviposition estimated by the forecasting model resulted in *r*_*s*_ = 0.80 (P < 0.05) (Fig 3).

## Discussion

The present study complements our earlier consideration of prevention of dengue outbreaks in Orán, which generated forecasts based on *Ae*. *aegypti*’s intrinsic rate of population growth [18]. The results of this study provide evidence that the *Ae*. *aegypti* oviposition dynamic is strongly influenced by meteorological variables, especially minimum temperatures required for the onset of oviposition. Increases in the minimum critical temperature (over 10°C) are associated with an increase in vector oviposition activity 3 weeks later. Secondly, a three week lag period after rains (with adequate minimum temperatures) corresponds with an ensuing increase in oviposition. This 3 week lag period corresponds well with the time required for larval development, pupation and adult oogenesis.

*Aedes aegypti* oviposition activity varied seasonally in Orán. This variation probably led to variations in adult abundance as suggested by Micieli et al. [31]. During the first year of the study, female oviposition activity was continuous, although no eggs were found during winter of the second year (July-September), when temperatures were lower than the normal for that period (considering records of the last 20 years). An abnormally lengthy polar wave covered the north of the country, resulting in snowfall in temperate zones and frosts in subtropical areas (SMN). Furthermore, the temperature anomalies registered during winter 2007 apparently were affected by an El Niño-type sea level pressure pattern across the equatorial Pacific basin [32].

The thermal threshold below which no eggs were found occurred when temperatures dropped below 17°C for Córdoba [33] and Quilmes, Buenos Aires province [34], whereas 13°C was the apparent threshold in La Plata City, Buenos Aires province [31]. In our study, no eggs were found when the temperature dropped below 10°C. De Garín et al. [35] pointed out that an increase in the minimum temperature could have a significant effect on *Ae*. *aegypti* abundance in Buenos Aires, just as our data have shown.

Discontinuous oviposition activity during the second sampling year in Orán (unusual due to the subtropical conditions, but with one or two frosts per year), can be compared with the fluctuation patterns observed in temperate areas of the country, with strong seasonality, as shown in Córdoba [33], Buenos Aires [36], and Quilmes (Buenos Aires Province) [34]. In temperate areas, the seasonal abundance of the vector could be conditioned by the environmental temperature which would stop the activity in winter. First spring females derive from overwintering eggs, reinitiating the vector cycle once again [33].

Our findings highlight the importance of minimum temperatures in vector activity, as shown by the lack of oviposition during winter of 2007. The time lag with the best correlation was 3 weeks, which means that increases in the minimum critical temperature (over 10°C) are associated with an increase in the vector oviposition activity 3 weeks later. For Córdoba, in temperate Argentina, the best time lag for temperature was 4 weeks [33]. For *Ae*. *aegypti* larval indices in Orán, the best correlation with the minimum temperatures was registered 4 weeks before [16]. Furthermore, minimum temperature was found to be among the important meteorological predictors for *Ae*. *aegypti* dynamics in Ecuador [37]. Minimum temperature seems to be critical in many regions for the threshold of mosquito survival and development to sustain the population density [38].

Estallo et al. [18] found that *Ae*. *aegypti* oviposition activity was continuous (but low) during dry winters, whereas it was higher during the rainy season and increased with rainfall intensity. Similar results were obtained by Dibo et al. [39], who related an adult and egg abundance of *Ae*. *aegypti* with frequency and intensity of rainfall in Mirasol, Brazil. With a time lag of 3 weeks, rainfall was one of the variables that best correlated with the number of eggs, confirming its importance in the study area. Something similar was observed in Orán with the larval indices, where an increase in such indices was preceded 4 weeks in advance by an increase of rainfalls [16]. An increase in oviposition activity 4 weeks after the increase of rainfalls was also detected in Córdoba [33] and, a 3 week lag was significant in Ecuador [37]. We could expect that an increase in oviposition of *Ae*. *aegypti* in Orán should occur 3 weeks after rains with adequate minimum temperatures.

The high number of eggs observed during January, February and March are probably due to the rainfall that occurred 3 weeks before and the availability of artificial containers around houses (personal observation). In this study, the peak in the number of eggs registered during December 2006 was clearly associated with rains in November 2006, which were 100% higher than the rainfall registered in the same period in 2005. In March, there was another peak of precipitation which did not involve an increase in the number of eggs, probably because in April, at the beginning of autumn, there was a decrease in temperature, as well as humidity and high vapor pressure of water, relative to summer values.

The water vapor pressure showed better correlation (*r*_*s*_ = 0.76) without time lag, suggested that this meteorological variable affects directly the egg laying activity of females. It is calculated as a function of temperature and humidity. When there is a decrease in water vapor pressure, a decline in the number of eggs is registered, probably because adult mortality increased, and therefore decreased oviposition activity [40]. Micieli and Campos [41], for northwestern locations of Argentina, found that in winter the decline in the population of *Ae*. *aegypti* could be correlated with a reduction in relative humidity. Similarly, we found that a decrease in the number of collected eggs was accompanied by a decrease in the water vapor pressure, and consequently, in relative humidity (though the observed correlation values between humidity and number of collected eggs were lower with and without time lags than the correlation observed between the number of collected eggs and water vapor pressure).

Humidity showed a low correlation with the number of eggs collected, although it was included by the predicting model. This would indicate that this variable alone does not affect *Ae*. *aegypti* activity as it does when in combination with temperature. Humidity had its best correlation with a time lag of 2 weeks, a value consistent with a study in Queensland, Australia that correlated humidity with *Ae*. *aegypti* adult abundance [42], and another in Orán which correlated humidity with larval indices [16].

For maximum temperatures, the best time lag found in this study was 6 weeks. These results are similar to those found by Dibo et al. [39] in Mirasol, Brazil, with average temperatures that rose 7 weeks before the increase in the abundance of *Ae*. *aegypti* adults and eggs.

## Conclusions

Our model predicts an increase in oviposition of *Ae*. *aegypti* in Orán 3 weeks after rains during periods with minimum temperatures (over 10°C). We anticipate that such a model will be most useful as an early warning tool for predicting peaks of activity by the vector population. A more localized and comprehensive analysis using site-specific data necessary for an effective disease prevention program [39]. Additional variables, including social factors (demographic, water access and storage, knowledge and perceptions of the vector and dengue disease, housing conditions) are not included here and may also add to predictability.
